# A Long-Term Treatment with Arachidonyl-2′-Chloroethylamide Combined with Valproate Increases Neurogenesis in a Mouse Pilocarpine Model of Epilepsy

**DOI:** 10.3390/ijms18050900

**Published:** 2017-04-25

**Authors:** Marta Andres-Mach, Mirosław Zagaja, Agnieszka Haratym-Maj, Radosław Rola, Maciej Maj, Joanna Haratym, Monika Dudra-Jastrzębska, Jarogniew J. Łuszczki

**Affiliations:** 1 Isobolographic Analysis Laboratory, Institute of Rural Health, Jaczewskiego 2, 20-950 Lublin, Poland; lasius1981@wp.pl (M.Z.); jarogniew.luszczki@gmail.com (J.J.Ł.); 2 Department of Physiopathology, Institute of Rural Health, Jaczewskiego 2, 20-950 Lublin, Poland; agamaj3@poczta.onet.pl (A.H.-M.); rola.radoslaw@gmail.com (R.R.); mdudra@wp.pl (M.D.-J.); 3 Department of Neurological Surgery, Medical University of Lublin, Jaczewskiego 8, 20-090 Lublin, Poland; 4 Department of Biopharmacy, Medical University of Lublin, Chodzki 4A, 20-090 Lublin, Poland; ewolucjonista@gmail.com; 5 Department of Anestesiology and Intensive Care Medicine, Hollycross Cancer Center, Artwińskiego 3, 25-734 Kielce, Poland; harunia@poczta.onet.pl; 6 Department of Pathophysiology, Medical University of Lublin, Jaczewskiego 8, 20-090 Lublin, Poland

**Keywords:** ACEA, valproate, neurogenesis, pilocarpine, seizures

## Abstract

Rational polytherapy in the treatment of refractory epilepsy has been the main therapeutic modality for several years. In treatment with two or more antiepileptic drugs (AEDs), it is of particular importance that AEDs be selected based on their high anticonvulsant properties, minimal side effects, and impact on the formation of new neurons. The aim of the study was to conduct an in vivo evaluation of the relationship between treatments with synthetic cannabinoid arachidonyl-2′-chloroethylamide (ACEA) alone or in combination with valproic acid (VPA) and hippocampal neurogenesis in a mouse pilocarpine model of epilepsy. All studies were performed on adolescent male CB57/BL mice with using the following drugs: VPA (10 mg/kg), ACEA (10 mg/kg), phenylmethylsulfonyl fluoride (PMSF—a substance protecting ACEA against degradation by fatty acid hydrolase, 30 mg/kg), pilocarpine (PILO, a single dose of 290 mg/kg) and methylscopolamine (30 min before PILO to stop peripheral cholinergic effects of pilocarpine, 1 mg/kg). We evaluated the process of neurogenesis after a 10-day treatment with ACEA and VPA, alone and in combination. We observed a decrease of neurogenesis in the PILO control group as compared to the healthy control mice. Furthermore, ACEA + PMSF alone and in combination with VPA significantly increased neurogenesis compared to the PILO control group. In contrast, VPA 10-day treatment had no impact on the level of neurons in comparison to the PILO control group. The combination of ACEA, PMSF and VPA considerably stimulated the process of creating new cells, particularly neurons, while chronic administration of VPA itself had no influence on neurogenesis in the mouse pilocarpine model of epilepsy. The obtained results enabled an in vivo evaluation of neurogenesis after treatment with antiepileptic drugs in an experimental model of epilepsy.

## 1. Introduction

Temporal lobe epilepsy (TLE) is the most common type of partial or localization-related epilepsy. Until recently, epilepsy treatment was mainly aimed at stopping seizures. However, for the past several years many researchers have focused their efforts on searching for new, potent anticonvulsant, natural or synthetic, which will not only stop seizures, but also have neuroprotective properties and no side effects [1,2,3,4,5]. Because human TLE is the most common type of epilepsy, animal models of these conditions are thought to be best in helping us understand the problem of epileptogenesis and the neuronal alterations taking place in a given region of the brain after convulsions [6].

Hippocampal neurogenesis is very sensitive to many different physiological and abnormal impulses. Epileptic seizures should be distinguished among the most common stimuli, as they change not only the extent, but also the pattern of neurogenesis. In addition to seizures, antiepileptic drugs also have a significant impact on neurogenesis [7,8,9,10,11]. One of the well-known first-line antiepileptic drugs is valproic acid (VPA). An overview of in vitro/in vivo studies regarding VPA and its impact on convulsions, neuroprotection and neurogenesis often returns contradictory results [12,13,14]. Umka et al. [15] revealed that VPA can cause cognitive impairment, which is associated with changes in hippocampal neurogenesis and neurotrophin levels in rats. Interestingly, recent studies on *Xenopus laevis* tadpoles indicated that VPA induces abnormal visual avoidance and schooling behaviors [16].

It has already been shown in many animal models of epilepsy that the endocannabinoid system plays a critical role in modulating seizure activity [4,5,6,7,8,9,10,11,12,13,14,15,16,17,18,19]. We have studied synthetic cannabinoid arachidonyl-2′-chloroethylamide (ACEA) alone and in combination with different antiepileptic drugs (AEDs) in various animal models of epilepsy. Luszczki et al. [2] showed an enhanced anticonvulsant activity of phenobarbital caused by ACEA and phenylmethylsulfonyl fluoride (PMSF), a lack of pharmacokinetic interaction and no acute adverse effects of the examined compounds in the mouse maximal electroshock seizure model (MES). Subsequently, research using the mouse pentylenetetrazole (PTZ)-induced clonic seizure model revealed that ACEA significantly potentiated the anticonvulsant activity of VPA. It should be emphasized that ACEA with VPA did not affect motor coordination in the chimney test, long-term memory in the passive avoidance task, or muscular strength in the grip-strength test in mice, indicating no possible acute adverse effects in animals [18]. Moreover, Florek-Luszczki et al. [20] indicated that ACEA clearly enhanced the anticonvulsant potency of pregabalin in the MES test by significantly decreasing the median effective dose of pregabalin.

The endogenous cannabinoid system seems to be very important in the modulation of adult neurogenesis [21,22,23]. The results obtained in our last neurogenesis study of ACEA and VPA chronic treatment in CB57/BL mice indicated a significant impact of this synthetic cannabinoid (administered alone and in combination with VPA) on proliferation of newborn cells [11].

On the basis of the results obtained from PTZ-induced clonic seizure model [18], as well as our recent neurogenesis study, we hypothesized that both the combination of ACEA + VPA and VPA administered alone may have an impact on neurogenesis in the pilocarpine model of epilepsy in mice. To confirm our assumptions, we decided to conduct an in vivo evaluation of the relationship between treatment with synthetic cannabinoid ACEA in combination with VPA and hippocampal neurogenesis in the mouse pilocarpine model of epilepsy.

## 2. Results

### 2.1. The Effect of Pilocarpine on Proliferation of Newborn Cells

**Figure 1:**
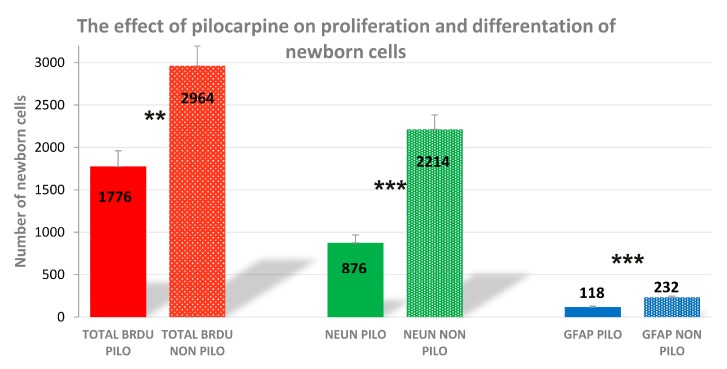
The numbers of cells represent an estimate of the total number of positively labeled cells in the subgranular zone in both hemispheres. The results were analyzed using Student’s *t*-test. Each bar represents the mean for five mice; error bars are the standard error of the mean (S.E.M., ** *p* < 0.01; *** *p* < 0.001).

The obtained results indicated a decrease of neurogenesis in the pilocarpine (PILO) control group in comparison to the results of control healthy mice obtained from our previous research [11]. As we reported, in the control healthy group the total number of bromodeoxyuridine (BrDU) positive cells in the dentate gyrus of mice averaged 2964 ± 232, while the results from the PILO studies indicated that in the PILO control group the total number of BrdU positive cells in the dentate gyrus of mice averaged 1776 ± 150 (*t*_8_ = 4.332; *n* = 5; *p* = 0.0025; Figure 1). Similarly, a significant difference in the amount of double stained NeuN/BrdU positive cells was observed between the healthy control and PILO control groups (876 ± 74 and 2214 ± 170 respectively; *t*_8_ = 7.084; *n* = 5; *p* = 0.0001; Figure 1). Additionally, the average number of GFAP/BrdU positive cells for control healthy mice was 232 ± 14, whereas for the PILO control group it was 118 ± 10 (*t*_8_ = 6.110; *n* = 5; *p* = 0.0003; Figure 1).

### 2.2. The Impact of Synthetic Cannabinoid Arachidonyl-2′-Chloroethylamide (ACEA) and Valproic Acid (VPA) on Total Newborn Cells in the Dentate Subgranular Zone of Pilocarpine (PILO) Mice

**Figure 2:**
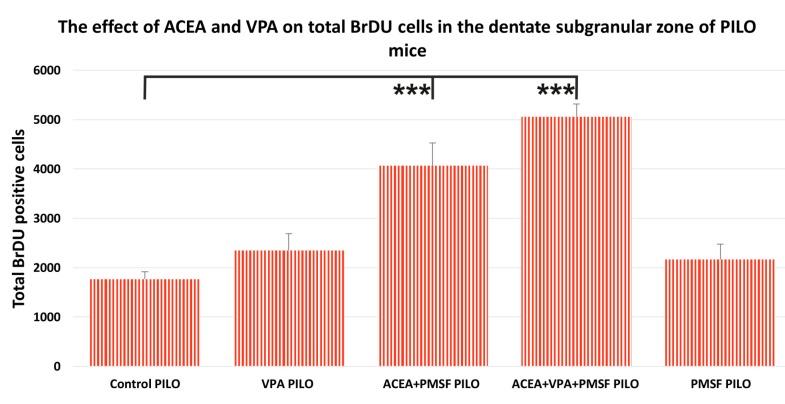
The numbers of cells represent an estimate of the total number of positively labeled cells in the subgranular zone in both hemispheres. The results were analyzed using one-way analysis of variance (ANOVA) followed by Dunnett’s test for multiple comparisons. Each bar represents the mean for five mice; error bars are S.E.M. (*** *p* < 0.001).

The results from the neurogenesis study indicated that the combination of ACEA + PMSF + VPA PILO as well as ACEA + PMSF PILO increased the total number of BrdU positive cells as compared to the control PILO group (Figure 2). As mentioned above, in the control PILO group the total number of BrdU positive cells in the dentate gyrus of mice averaged 1776 ± 150, while in ACEA + PMSF + VPA PILO mice the average value was 5056 ± 259 (*F*_4,23_ = 21.53; *n* = 5; *p* < 0.001; Figure 2), and for ACEA + PMSF PILO mice the average value was 4068 ± 457 (*p* < 0.001 for comparisons). No statistical significance was observed when comparing VPA PILO to the control PILO group (*p* > 0.05 for comparisons). Similarly, the total number of BrdU positive cells in PMSF PILO mice had no significant difference as compared to the control PILO group (*p* > 0.05 for comparisons).

### 2.3. The Impact of ACEA and VPA on Newborn Neurons in the Dentate Subgranular Zone of PILO Mice

**Figure 3:**
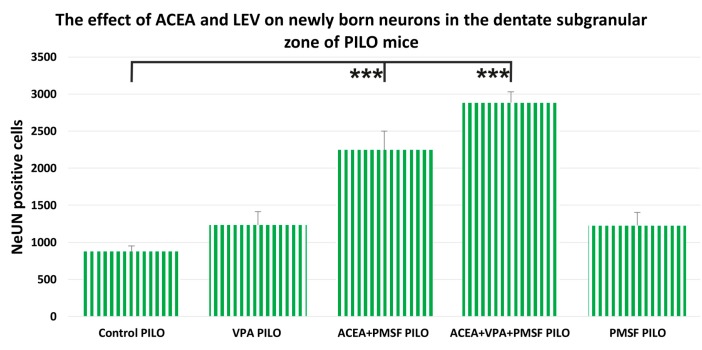
The effects of synthetic cannabinoid arachidonyl-2′-chloroethylamide (ACEA) and valproic acid (VPA) on newly born neurons in the dentate subgranular zone of PILO mice. The numbers of cells represent an estimate of the total number of positively labeled cells in the subgranular zone in both hemispheres. The results were analyzed using one-way analysis of variance (ANOVA) followed by the Dunnte’s test for multiple comparisons. Each bar represents the mean for five or six mice; error bars are S.E.M. (*** *p* < 0.001).

In the control PILO group, the number of BrdU positive cells colocalized with NeuN in the dentate gyrus of mice averaged 876 ± 74, while in ACEA + PMSF PILO-treated mice the average was 2246 ± 252 (*F*_4,23_ = 25.4; *n* = 5; *p* < 0.001; Figure 3), and 2882 ± 147 in ACEA + PMSF + VPA PILO-treated mice (*p* < 0.001 for comparisons). The total number of NeuN/BrdU positive cells in PMSF PILO mice was not significantly different as compared to the control PILO group (*p* > 0.05 for comparisons). VPA PILO mice showed a slight increase of NeuN/BrdU cells when compared to the control PILO group, but the difference was not statistically significant (*p* > 0.05 for comparisons).

### 2.4. The Impact of ACEA and VPA on Newborn Astrocytes in the Dentate Subgranular Zone of PILO Mice

**Figure 4:**
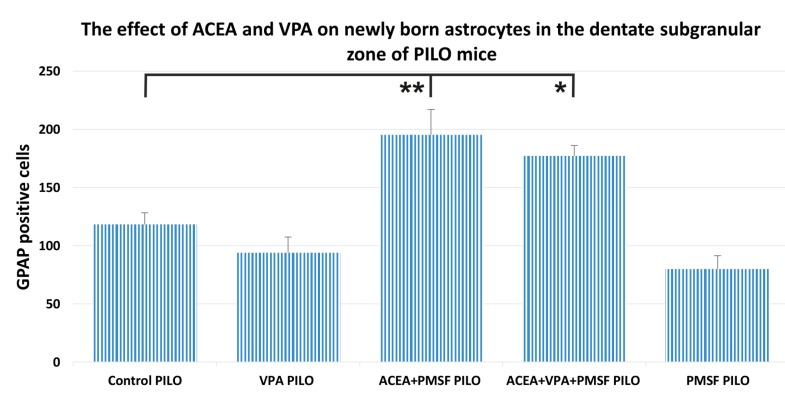
The effects of ACEA and VPA on newly born astrocytes in the dentate subgranular zone of PILO mice. The numbers of cells represent an estimate of the total number of positively labeled cells in the subgranular zone in both hemispheres. The results were analyzed using one-way analysis of variance (ANOVA) followed by the Dunnet’s test for multiple comparisons. Total numbers of BrdU/GFAP-positive cells slightly decreased after VPA PILO injections, whereas in ACEA + PMSF + VPA PILO-treated mice a significant increase in newly born cells was observed as compared to the PILO control group (*p* < 0.05). The total number of BrdU/GFAP positive cells in PMSF PILO mice was not significantly different in comparison to the PILO control group (*p* > 0.05 for comparisons). Each bar represents the mean for five or six mice; error bars are S.E.M. (* *p* < 0.05; ** *p* < 0.01).

Both in the ACEA + PMSF + VPA PILO and ACEA + PMSF PILO group, a significant impact on newborn astrocytes was revealed (Figure 4) as compared to the control PILO mice. The average number of astrocytes for control PILO mice was 118 ± 10, whereas for ACEA + PMSF PILO-treated mice it was 195 ± 22, and the number of GFAP/BrdU positive cells for ACEA + PMSF + VPA PILO-treated mice averaged 177 ± 9 (*F*_4,23_ = 14.39; *p* < 0.01, *p* < 0.05, respectively; *n* = 5; Figure 4). VPA PILO mice showed a slight increase in GFAP positive cells when compared to the control PILO group, but the difference was not statistically significant (*p* > 0.05 for comparisons). The total number of GFAP positive cells in PMSF PILO mice was not significantly different compared to the control PILO group (*p* > 0.05 for comparisons).

## 3. Discussion

The results we obtained from this in vivo study showed a difference in the level of neurogenesis between healthy control mice and control mice with spontaneous seizures induced by pilocarpine injection. Moreover, we indicated that ACEA (10 mg/kg, i.p.) co-administered with PMSF (30 mg/kg, i.p.) significantly changed the total number of BrdU positive cells in PILO mice. The combination of ACEA + PMSF + VPA significantly enhanced BrdU positive cells of PILO mice. A 10-day treatment with VPA showed no significant influence on the process of neurogenesis as compared to the control PILO group. However, it should be emphasized that neurogenesis in the PILO control group is strongly decreased as compared to healthy control mice.

Neurogenesis persists throughout adulthood in mammals, specifically in the subgranular zone of the hippocampal dentate gyrus and the subventricular zone (SVZ) of the forebrain lateral ventricles [24]. Endogenous neural stem cells are known to substitute lost neurons in the adult brain, which may reduce the negative effects of patients with chronic neurodegenerative diseases including epilepsy [25]. However, such a neurogenesis may be harmful and could foster the progression of seizures. Aberrant maturation of newborn neurons may play a role in the development of chronic epileptic seizures [26]. Although acute seizures lead to an increase of proliferation of newborn cells, hippocampal neurogenesis is reduced at chronic stages of epilepsy [27]. Moreover, treatment with antiepileptic drugs may also have various impact on neurogenesis [7,8,9,10].

VPA is used primarily to treat epilepsy and bipolar disorders, but also to prevent migraine headaches [28,29,30,31,32]. It has been demonstrated in various animal models of epilepsy that VPA enhances its antiepileptic activity in combination with many natural and synthetic substances that have potential anticonvulsant properties [4,33,34]. Although VPA is a commonly used antiepileptic drug worldwide, its toxicity and teratogenicity is a relevant problem especially in the treatment of women at childbearing age [35]. Prenatal VPA exposure of neuronal cultures from the cerebral cortices of prenatal mice embryos was shown to decrease the total number, total length, and complexity of neuronal dendrites [36]. Similarly, results obtained by Semmler et al. [37] indicated that intrauterine VPA exposure caused dose-dependent neuronal cell number alterations in the hippocampal areas CA1/2 and the CA3 region and in folic acid metabolism in a rat model of valproate teratogenicity. On the other hand, VPA appears to cooperate in neuroprotection and cognitive enhancement by inhibition of histone deacetylase (HDAC) activity [38]. Furthermore, VPA has been shown to have neuroprotective properties in traumatic brain injury (TBI), Alzheimer’s disease, Parkinson’s disease, Huntington’s disease and amyotrophic lateral sclerosis [39,40,41,42,43,44]. Taking into consideration the impact of VPA on the process of proliferation, migration and differentiation of newborn cells, we can find many different and contradictory results. Kim et al. [45] showed that BrdU administration followed by one week of VPA injection resulted in a small increase in the survival and phenotypical differentiation of maturing neurons. On the other hand, VPA injected for ten days reduced proliferation (Ki-67), with no significant reduction in doublecortin (DCX) levels within the hippocampus of rats [15]. In our previous studies, we have shown that VPA decreased the proliferation and differentiation of newborn cells, but without statistical significance when compared to the control group, thus demonstrating that VPA affects neurogenesis in healthy mouse brains [11].

Results from various VPA studies reveal that this antiepileptic drug has many effects on neurogenesis depending on the type of investigation (acute/chronic, in vivo/in vitro studies). The results obtained from a study using a chronic dietary administration of VPA following BrdU injection in mice showed an enhanced proliferation in the hippocampal dentate gyrus [28]. In their studies, Vukićević et al. [46] focused on the impact of VPA on the epigenetic effects in two culture conditions: sympathoadrenal progenitors within free-floating chromospheres and adherent cell cultures optimized to derive neurons. The results they obtained indicated that VPA launches differentiation mechanisms in sympathoadrenal progenitor cells that result in an increased generation of functional neurons.

Postnatal cognitive functional impairment after prenatal VPA exposure in mice caused by the untimely enhancement of embryonic neurogenesis led to the depletion of neural precursor cells pool and, consequently, a decreased level of adult neurogenesis in the hippocampus [47]. However, it turns out that these impairments can be alleviated by voluntary running.

Very interesting findings regarding the effects of VPA on proliferation were reported by Boku et al. [48], who used adult dentate gyrus-derived neural precursor cells isolated from adult male Sprague–Dawley rats. VPA used in this study significantly increased the ratio of astrocytes and decreased the level of neurons. On the other hand, results from the study on cultured adult spinal neural stem/precursor cells (NSPCs) from chronic compressive spinal cord injury (SCI) rats treated with VPA showed that the administration of VPA arrested proliferation, but promoted neuronal differentiation of spinal NSPCs [49].

One of the main reasons for such different results is the fact that VPA has many paths of action and a variety of molecular mechanisms involved in the regulation of neuronal processes. We should also take into account additional factors such as the dose, various models of investigation, as well as time and route of drug administration, which undoubtedly influence the regulation of neuronal processes [38].

Despite the fact that a number of antiepileptic drugs are known and commonly used, scientists are still looking for new substances with antiepileptic but also neuroprotective properties. According to recent studies, cannabinoids and the endocannabinoid system were consistent with the profile of such research.

Only in the past few years ACEA, one of the best known and studied synthetic cannabinoids, has been shown to have strong antiepileptic properties in various in vivo studies: pentylenetetrazole (PTZ) model of myoclonic seizures in mice [18,50,51], the maximal electroshock seizure model in mice [2,19], penicillin-induced epileptiform activity in rats [52,53,54]. Apart from ACEA, some other cannabinoids have been studied for neurobiological properties. Jiang et al. [55] indicated that chronic treatment with synthetic cannabinoid HU-210 promoted neurogenesis in the hippocampal dentate gyrus of adult rats and exerted anxiolytic- and antidepressant-like effects. Selective stimulation of CB_1_ and CB_2_ receptors using ACEA and JWH133 was shown to counteract the alcohol-induced decrease in NPC proliferation in the brain of adult rats with a forced consumption of alcohol [56]. In turn, results obtained from the treatment with cannabinoids WIN 55,212-2 and 2-arachidonoylglycerol (2-AG) in the mouse olfactory epithelium in vivo indicated increased proliferation, but not neurogenesis nor non-neuronal cell generation [57]. Additionally, Vinogradowa and van Rijn [58] examined acute and long-term effects of another synthetic cannabinoid WIN55,212-2 in the early stage of audiogenic kindling. The results they obtained showed that WIN55,212-2 administered in a single dose one hour before the 4th seizure delayed the kindling process by two weeks, without any acute effect on audiogenic seizures.

The results from our last study concerning the evaluation of the impact of ACEA administered alone and in combination with VPA on the proliferation and differentiation of neural precursor cells in the mouse brain clearly indicated that ACEA in combination with VPA increases the number of Ki-67-positive cells in mice. Moreover, ACEA administered alone and in combination with VPA significantly increases the level of total BrdU positive cells as well as newborn neurons and astrocytes, which confirms its impact on neurogenesis [11]. Moreover, we indicated a significant increase in NeuN positive cells for ACEA + PMSF and ACEA + PMFS + VPA versus VPA-treated mice. A similar effect of the protection of neurogenesis was observed by Welbat et al. [59]. They found that a long-term treatment (2 weeks) with VPA (300 mg/kg) causes impairments of spatial working memory, cell proliferation and survival in the subgranular zone (SGZ) of the hippocampal dentate gyrus (DG) in Spraque-Dawley rats, but oral administration of Asiatic acid (30 mg/kg/day) for 28 days clearly prevented spatial memory and neurogenesis impairments caused by VPA. Moreover, in a subsequent study they showed that *Kaempferia parviflora*, a herbal plant whose rhizomes are used in traditional medicine, prevents the cognitive decline and reduction in proliferating cells caused by VPA. Additionally, co-treatment significantly increased DCX protein levels within the hippocampus. The obtained results indicate that *K. parviflora* is able to prevent the VPA-induced impairments of spatial memory and the proliferation of cells within the SGZ [60].

## 4. Materials and Methods

### 4.1. Animals and Experimental Conditions

Adolescent male CB57/BL mice (6 weeks old) were obtained from Mossakowski Medical Research Centre, Polish Academy of Sciences, Warsaw, Poland. The mice were kept in colony cages with free access to food and tap water ad libitum, under standardized housing conditions (a natural light-dark cycle, a temperature of 22–24 °C). After 7 days of adaptation to laboratory conditions, the animals were randomly assigned to experimental groups consisting of eight mice. Each mouse was used only once. All tests were performed between 9.00 a.m. and 2.00 p.m. Procedures involving animals and their care were conducted in conformity with current European Community and Polish legislation on animal experimentation. Additionally, all efforts were made to minimize animal suffering and to use only the number of animals necessary to produce reliable scientific data. The experimental protocols and procedures listed below were also in accordance with the Guide for the Care and Use of Laboratory Animals and were approved by the Local Ethics Committee at the University of Life Sciences in Lublin (License No.: 23/2013, Date: 12 March 2013)

### 4.2. Drugs

The following drugs were used in this project: valproate sodium (VPA, kindly donated by ICN Polfa S.A., Rzeszow, Poland); arachidonyl-2′-chloroethylamide or *N*-(2-chloroethyl)-5*Z*,8*Z*,11*Z*,14*Z*-eicosatetraenamide, pre-dissolved in anhydrous ethanol (ACEA; Tocris Cookson Ltd., Bristol, UK); phenylmethylsulfonyl fluoride (PMSF; ICN Biomedicals Inc., Irvine, CA, USA); pilocarpine (MP Biomedicals, Illkirch-Graffenstaden, France); methylscopolamine (Sigma Aldrich, Schnelldorf, Germany). PMSF was used to limit the degradation of ACEA by inhibiting fatty acid amide hydrolase [4]. VPA and ACEA were dissolved in distilled water. All drugs were administered intraperitoneally (i.p.) in a single injection, at a volume of 0.005 mL/g.

### 4.3. Pilocarpine-Induced Convulsions

The mice were housed individually on a 12-h day/night cycle at least 7 days prior to the treatment with free access to food and tap water ad libitum. Pilocarpine study was performed in accordance to the procedure described by Bahaskaran and Smith [61] with minor changes. Taking into account the possibility of 20% mortality as well as lack of seizures after pilocarpine administration, 60 mice were included in the study (to obtain at least 8 PILO mice per group). Mice were administered an i.p. injection of methylscopolamine (1 mg/kg) 30 min prior to the injection of pilocarpine to reduce the peripheral cholinergic effects of pilocarpine. Experimental animals were then injected i.p. with a single dose of pilocarpine of 290 mg/kg [62]. The mice were carefully monitored after the pilocarpine injection to observe the first symptoms of convulsions. Seizure behavior occurred about 2 h after the pilocarpine injection and was evaluated according to Racine’s 1–5 scale [63,64]. The most important were convulsive seizures (categories 3 to 5), which correlate with the eventual development of spontaneous seizures and mossy fiber sprouting. A mouse that experienced a minimum of 3 generalized convulsive seizure events within 2 h following the pilocarpine injection was considered to have undergone status epilepticus (SE). The category 3–5 of spontaneous seizures was assessed by passive observation of 2 h/day, for one week after SE. The animals with spontaneous seizures (PILO mice) were used for the next step of the experiment. For the animals in which no seizures were observed, euthanasia with carbon dioxide inhalation was performed.

### 4.4. Drug Administration

A week after SE, the PILO mice were treated with antiepileptic drugs for the next 10 days. To determine any changes in neurogenesis, the mice were divided into five groups: ACEA + PMSF; ACEA + PMFS + VPA; VPA; PMSF; Control (0.9% NaCl solution).

**Figure 5:**
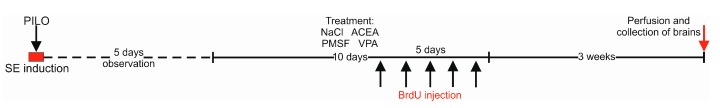
Schematic illustration of the experimental design used in the study. Experimental procedure: PILO—pilocarpine injection; SE—status epilepticus; ACEA—arachidonyl-2′-chloroethylamide; PMSF—phenylmethylsulfonyl fluoride; VPA—valproic acid; BrdU-5-Bromo-2-Deoxyuridine.

Fresh drug solutions were prepared ex tempore each day of the experiment and administered once a day at the following doses: VPA—10 mg/kg, ACEA—10 mg/kg, PMSF—30 mg/kg. The doses for ACEA and PMSF were chosen based on information about their efficacy from the PTZ-induced seizure model [18], where ACEA at a dose of 10 mg/kg in combination with VPA showed anticonvulsant effects and no learning or memory disturbances in the passive avoidance task. Additionally, BrdU (a marker of cell proliferation, 50 mg/kg) was given as one more single injection for the last 5 days of the 10-day treatment. For a detailed schematic illustration of the experiment design, please refer to Figure 5.

### 4.5. Tissue Preparation

Three weeks after the last BrdU injection, the mice were anesthetized and perfused with ice-cold saline followed by freshly prepared, ice-cold 4% paraformaldehyde (PFA) in PBS. The brains were removed, post-fixed in fresh 4% PFA for 24 h, and, subsequently, 50 µm coronal sections were cut using a vibratome (VT1000S, Leica Biosystems, Wetzlar, Germany).

### 4.6. Immunohistochemical Staining

Immunohistochemical staining was performed in accordance with the method described in our earlier study [11]. Fifty-micrometer sections were stored at 4 °C in cryoprotectant until needed. Free floating sections were immunostained using antibodies listed in Table 1.

### 4.7. Confocal Microscopy and Cell Counting

**Figure 6:**
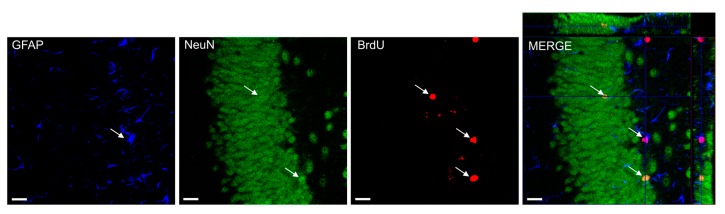
Bromodeoxyuridine (BrDU) positive cells in colocalization with NeuN and GFAP cells. The number of BrdU positive cells displaying astrocyte-specific (GFAP), neuron-specific (NeuN), BrDU-specific markers was determined using confocal microscopy to score the colocalization of BrdU and phenotypic indicators (MERGE-in orthogonal views) in representative sections from each animal. MERGE shows *Z*-axis projections of 23 μm × 1.32 μm. Bars: 20 μm.

Confocal imaging and quantitative analysis of newborn cells were performed according to the method described in our previous study [11]. To calculate the number of BrdU-positive (BrdU+) cells in the SGZ, at least 12 sections of a one-in-six series were scored per animal. All counts were limited to the dentate granule cell layer and a 50-μm border along the hilar margin that includes the SGZ. The total number of BrdU+ cells displaying neuron-specific (NeuN) or astrocyte-specific (GFAP) markers was determined using confocal microscopy to score the colocalization of BrdU and phenotypic indicators in representative sections from each animal (Figure 6). Confocal microscopy and cell counting were done using Zeiss LSM 5 Pascal microscope and ImageJ software. Appropriate gain and black-level settings were obtained on control tissues stained with secondary antibodies alone. Upper and lower thresholds were always set using a range indicator function to minimize data loss due to saturation. Each cell was manually examined in its full Z dimension using split panel analysis, and only those cells for which the BrdU-positive nucleus was unambiguously associated with the lineage-specific marker were scored as positive. For each lineage-specific marker, the percentage of BrdU-positive cells expressing the marker was determined [65]. The total numbers of lineage-specific BrdU-positive cells were calculated by multiplying this percentage by the total number of BrdU-positive cells in the dentate gyrus. The total numbers of respective cell types were obtained by multiplying the measured value by 6; overestimation was corrected using the Abercrombie method for nuclei with empirically determined average diameter of 13 μm within a 50-μm section [66].

### 4.8. Statistical Analysis

For each endpoint, values for all animals from a given treatment group were averaged and standard errors of mean (S.E.M.) were calculated. The results were analyzed using Student’s *t*-test and one-way Analysis of Variance (ANOVA) followed by the Dunnet’s test for multiple comparisons. The “*n*” in the presented study refers to the number of animals. All statistical tests were performed using the commercially available GraphPad Prism version 4.0 for Windows (GraphPad Software, San Diego, CA, USA).

## 5. Conclusions

It is evident that the endocannabinoid system has a significant impact on neurogenesis. The present study confirmed that a long-term treatment with an antiepileptic drug like VPA leads to a reduction of hippocampal proliferation as well as to migration and differentiation of newborn cells, whereas the use of the combination of ACEA and VPA significantly increases neurogenesis. Protection of the neurogenesis process certainly has great importance for epileptic patients undergoing long-term treatment with antiepileptic drugs, so an adjunctive antiepileptic therapy with a combination of ACEA is worthy of consideration in further preclinical trials.

## Figures and Tables

**Table 1:** Primary and secondary antibodies used in this study.

Target	Origin	Company	Cat. Number	Dilution
Neurons (NeuN)	Mouse	Millipore	MAB377	1:200
Mouse IgG	Goat	Jackson Immunoresearch	715-095-150	1:200
Astrocytes (GFAP)	Rabbit	DakoCytomation	Z033401	1:500
Rabbit IgG	Goat	Invitrogen	A-21071	1:200
S-phase cells (BrdU)	Rat	Accurate Chem	OBT0030S	1:10
Rat IgG	Donkey	Jackson Immunoresearch	712-295-153	1:200

## Abbreviations

TLE	Temporal lobe epilepsy
BrdU	5-Bromo-2-Deoxyuridine
NeuN	Neuronal nuclei
SGZ	Subgranular zone
GFAP	Glial fibrillary acidic protein
IP	Intraperitoneal
PMSF	Phenylmethylsulfonyl fluoride
PILO	Pilocarpine
ACEA	Arachidonyl-2′-chloroethylamide
VPA	Valproic acid
AEDs	Antiepileptic dgrus
PTZ	Pentylenetetrazole
MES	Maximal electroshock seizure
NSPCs	Neural stem/precursor cells
DCX	Doublecortin
